# The impact of intense nursing care in improving anxiety, depression, and quality of life in patients with liver cancer: A systematic review and meta-analysis

**DOI:** 10.1097/MD.0000000000021677

**Published:** 2020-08-21

**Authors:** Qiao Zhang, Rong Wan, Changdan Liu

**Affiliations:** a Department of Hepatobiliary Surgery; b Department of Midwifery, Jingzhou Central Hospital, Jingzhou, Hubei, China.

**Keywords:** anxiety, depression, intense nursing care, liver cancers, quality of life, self rating anxiety scale, self rating depression scale

## Abstract

**Background:**

Liver resection is a major, serious, and very delicate operation that should be done only by specialized, well-skilled, and experienced surgeons. However, the role of nurses, which has often been under-estimated, is also crucial for the success of the intervention or surgery. Intensive nursing care involves high quality nursing modes to achieve the expected goals of treatment smoothly and with less complications. In this analysis, we aimed to show the impact of intense nursing care in improving anxiety, depression, and quality of life in patients with intervention for liver cancers.

**Methods:**

Data sources included EMBASE, MEDLINE, Web of Science, the Cochrane central, Google scholar, and http://www.ClinicalTrials.gov. Three authors independently extracted data from the selected original studies. The statistical analysis was carried out by the Cochrane based RevMan software. For dichotomous data, the number of events and the total number of participants were required and for the continuous data, mean, standard deviation as well as the total number of participants were required in the input for analysis. Odds ratios (OR) with 95% confidence intervals (CI) were used to represent the data following assessment.

**Results:**

A total of 1205 participants with liver cancer enrolled between the years 2010 to 2018 were included in this analysis whereby 667 participants were assigned to an intensive nursing care. Our current analysis showed that most of the patients who were assigned to an intense nursing intervention were significantly very satisfied with their quality of life (OR: 4.07, 95% CI: 1.45 – 11.45; *P* = .008). However, a minor number of patients with liver cancer who were not assigned to intense nursing care were significantly dissatisfied with their quality of life with OR: 0.18, 95% CI: 0.04 – 0.77; *P* = .02. This analysis also showed that self-rating anxiety score (SAS) and self-rating depression score (SDS) were significantly in favor of the participants with intense nursing care with OR: − 7.66, 95% CI: [(−9.66) – (−5.66)]; *P* = .00001 and OR: −7.87, 95% CI: [(−8.43) – (−7.26)]; *P* = .00001 respectively. In addition, physical function (OR: 13.56, 95% CI: 12.39 – 14.74; *P* = .00001), and total activity score (OR: 16.58, 95% CI: 13.51 – 19.65; *P* = .00001) were also significantly in favor of an intense nursing care.

**Conclusions:**

Our current analysis showed that intense nursing care significantly improved anxiety, depression, and quality of life following interventions in patients with liver cancers. Most of the patients with liver cancers who were assigned to an intense nursing care were very satisfied with their quality of life. However, this hypothesis should further be confirmed in larger nursing related studies based on patients with liver cancers.

## 1 Introduction

Liver carcinoma is rapidly increasing in this new era with the increasing number of chronic hepatitis and liver cirrhosis. Statistics show that liver cancer accounts for about 42% of all cancers in China, and the number is rising drastically with approximately 600,000 new cases and 200,000 deaths annually.^[1]^ Hepatic surgeries and liver transplantations are the best options till date to treat liver cancers.^[2]^ According to the Organ Procurement and Transplantation Network, about 1000 liver transplantations have been done in the United States in 2016.^[3]^ However, whatever the treatment option, the decision should produce satisfactory outcomes in terms of survival and recurrence. For hepatocellular carcinoma patients whose tumors have successfully been resected, the 5-year survival rate lies between 10% to 60% depending on the type and size of the mass. The main goal of liver resection is to completely remove the tumor and the associated surrounding liver tissues with minimal residue and without inducing hepatic failure. However, in practice, because of these strict guidelines, only selected patients with liver cancer can undergo liver resection.^[4]^

Liver resection is a major, serious, and very delicate operation that should be done only by specialized, well-skilled, and experienced surgeons.^[5]^ However, this surgery also requires skillful and specialized nurses. The role of nurses have often been under-estimated and left unnoticed through research. It should be noted that intense care by nurses postoperatively has contributed immensely in the complete success of many surgeries.^[6]^ Patients are often anxious, depressed following hepatic surgeries, therefore psychological, and moral support are vital post-operatively to further improve the quality of life of these patients.

Intensive nursing care^[7]^ which has been defined as nursing care with additional caring facilities and methods including group nursing whereby several nurses are assigned to 1 particular patients, with combined nursing measures such as nursing plan, nursing philosophy, and nursing quality evaluation and which involves high quality nursing modes to achieve the expected goals of treatment smoothly and with less complications, should be implemented for these patients with intervention for liver cancers.

In this analysis, we aimed to show the impact of intense nursing care in improving anxiety, depression, and quality of life in patients with intervention for liver cancers.

## 2 Methods

### 2.1 Data sources and search strategies

Data sources included EMBASE, MEDLINE, Web of Science, the Cochrane central, Google scholar, and http://www.ClinicalTrials.gov.

Publications were searched using the following key terms:

- Nursing care and liver cancer;
- Nurse practice and liver disease;
- Nursing care and liver disease;
- Nurse intervention and liver cancer;
- Nurse intervention and liver surgery;
- Nurse intervention and hepatobilliary;

The term “liver” was also replaced by the word “hepatic” and the word “cancer” was substituted by “tumor”/”carcinoma”.

### 2.2 Inclusion and exclusion criteria

Studies were included if:

(a) They were trials or observational cohorts comparing participants with liver cancers who were assigned to an intense nursing care versus a control group;
(b) They reported potentially relevant endpoints including outcomes related to anxiety, depression, and quality of life;
(c) They were published in English language.

Studies were excluded if:

(a) They were review articles including meta-analysis/systematic reviews and literature reviews;
(b) They did not involve participants with liver cancers;
(c) They did not report the potentially relevant outcomes;
(d) They did not involve intense nursing care;
(e) They were duplicated studies which were repeated in different searched databases.

### 2.3 Definitions and Outcomes

Intensive nursing care has been defined as nursing care with additional caring facilities and methods including group nursing whereby several nurses are assigned to 1 particular patient, with combined nursing measures such as nursing plan, nursing philosophy, and nursing quality evaluation. It also involves high quality nursing modes to achieve the expected goals of treatment smoothly and with less complications.

Table 1 listed the endpoints which were reported in the original studies.

**Table 1:**
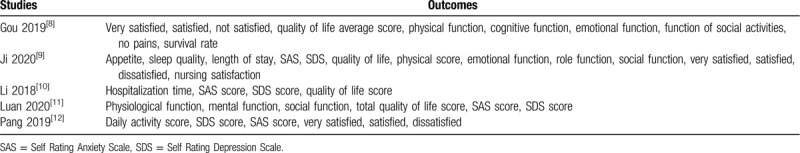
Outcomes reported in the original studies.

The final list of outcomes which were assessed in this meta-analysis was:

(a) Quality of life (including very satisfied, satisfied, and dissatisfied);
(b) Self-rating anxiety scale (SAS);
(c) Self-rating depression scale (SDS);
(d) Physical function;
(e) Total activity score;
(f) Social function.

### 2.4 Data extraction and quality assessment

Three authors independently extracted data from the selected original studies. The total number of participants with liver cancer who were assigned to intense nursing care and conventional nursing care were respectively extracted from each original studies. The total number of participants reporting their satisfaction with intense nursing care, the mean and standard deviation associated with SAS, SDS, physical function, total activity score, and social function were also carefully extracted to be used in the analysis. The time period of participants’ enrollment, the type of study, the year of publication, and the baseline features of the participants were also carefully extracted. Any disagreement which occurred during the data extraction process was resolved by a discussion with the corresponding author.

Two methodological assessment tools including the Cochrane collaboration^[13]^ for randomized trials and the Newcastle Ottawa scale (NOS)^[14]^ for observational studies were used for the quality assessment. Grades ranging from A to C were allotted to the studies, whereby a grade A denoted a low bias risk and a grade C denoted a high bias risk.

### 2.5 Statistical analysis

The statistical analysis was carried out by the Cochrane based RevMan software (Version 5.3).

For dichotomous data, the number of events and the total number of participants were required.

For the continuous data, the mean, standard deviation as well as the total number of participants were required in the input for analysis.

Odds ratios (OR) with 95% confidence intervals (CI) were used to represent the data following assessment by RevMan software.

The assessment of heterogeneity was carried out by the Q statistic test whereby a particular subgroup analysis showed a statistically significant result if the corresponding P value was less or equal to .05.

Assessment of heterogeneity was also done by observing the I^2^ value. The range of I^2^ value was reported to be between 0% to 100%. Heterogeneity increased with an increasing I^2^ value.

In this analysis, a random effects statistical model was used during assessment of the subgroups.

An exclusion method whereby each study was excluded one by one and a new analysis was carried out each time, was used for sensitivity analysis.

Publication bias was visually assessed through asymmetry of funnel plots which were generated through the RevMan software.

### 2.6 Ethical approval

This study is a meta-analysis and included data from previously published original studies. No experiment was carried out on animals or human beings by any of the authors. Therefore, an ethical approval was not required.

## 3 Results

### 3.1 Results of the search process

**Figure 1:**
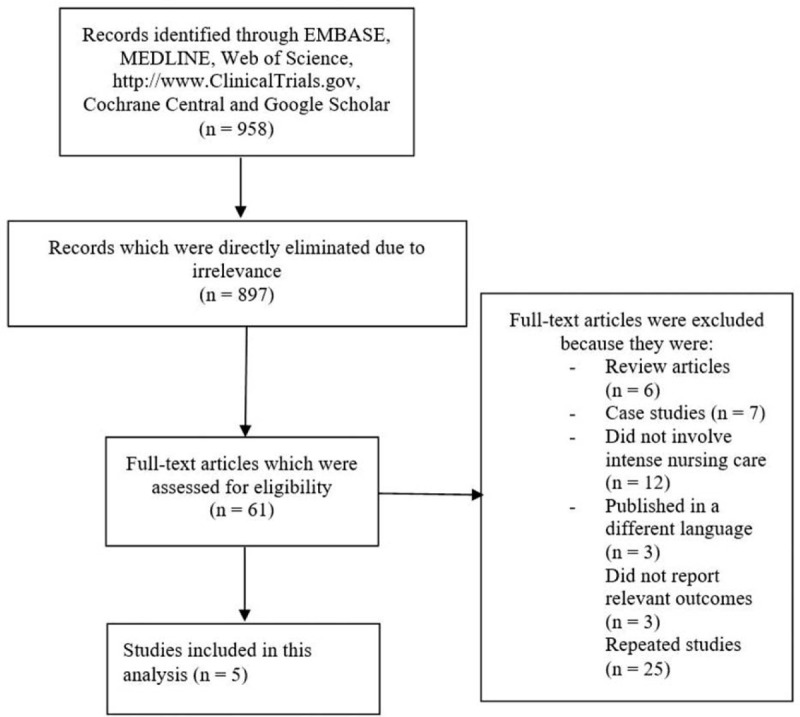
Flow diagram representing the study selection.

We followed the PRISMA reporting guideline for the search process.^[15]^ A total number of 958 articles were found through online databases. An initial assessment was carried out by the authors after having studied the titles and abstracts. Following this initial assessment, 897 articles were eliminated since they were not related to the topic of this research article. Sixty-one full text articles were assessed for eligibility. However, further elimination was carried out for the following reasons: review articles (6); case studies (7); did not involve intense nursing care (12); published in a different language apart from English (3); did not report the relevant outcomes (3); repeated studies (25). Finally, only 5 studies [8–12] were selected for this analysis as shown in Figure 1.

### 3.2 Main features of the selected studies

The main features of the original selected studies have been given in Table 2. Four studies were observational studies whereas 1 study was a randomized trial. A total of 1205 participants with liver cancer were included in this analysis whereby 667 participants were assigned to an intensive nursing care and 538 participants were assigned to a conventional nursing care as shown in Table 2. The time period of participants’ enrollment ranged from the year 2010 to year 2018.

**Table 2:**
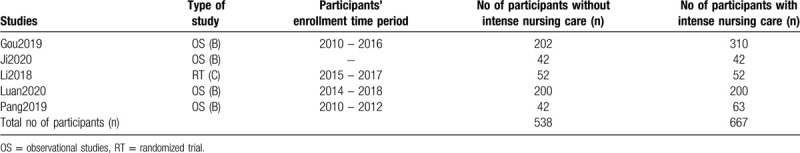
Main features of the participants.

After a methodological assessment of the studies, 1 study was allotted a grade C whereas the remaining 4 studies were allotted a grade B implying moderate bias risk as shown in Table 2.

### 3.3 Baseline features of the participants

The baseline features of the participants have been listed in Table 3. The mean age of the participants was 50.1 to 58.8 years. Male participants ranged from 54.76% to 87.13%. The percentage of patients who were smokers ranged from 47.62% to 70.0% as shown in Table 3.

**Table 3:**
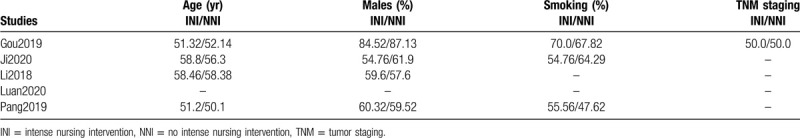
Baseline features of the studies.

### 3.4 Results of this analysis

**Figure 2:**
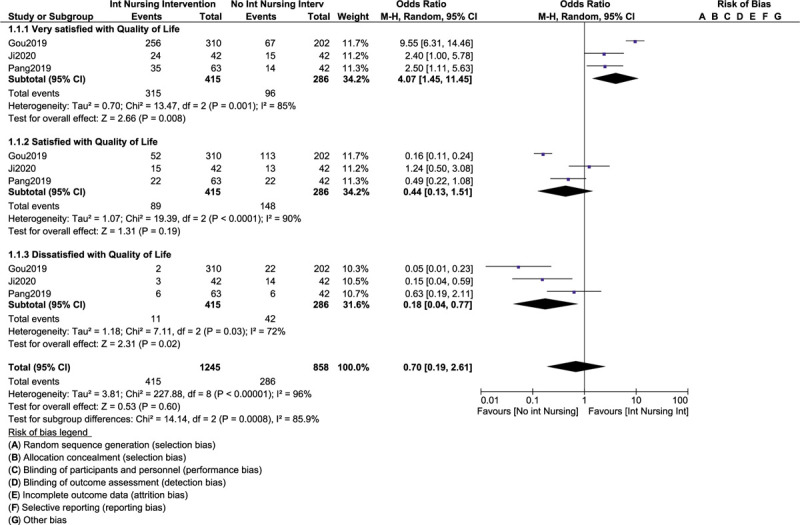
Improvement in quality of life following intense nursing care among patients with liver cancers.

Our current analysis showed that most of the patients who were assigned to an intense nursing intervention were significantly very satisfied with their quality of life (OR: 4.07, 95% CI: 1.45 – 11.45; *P* = .008) as shown in Figure 2. A minority of participants who were not assigned to an intensive nursing care was still satisfied with their quality of life (OR: 0.44, 95% CI: 0.13 – 1.51; *P* = .19). However, participants who were not assigned to intense nursing care were significantly dissatisfied with their quality of life (OR: 0.18, 95% CI: 0.04 – 0.77; *P* = .02) as shown in Figure 2.

**Figure 3:**
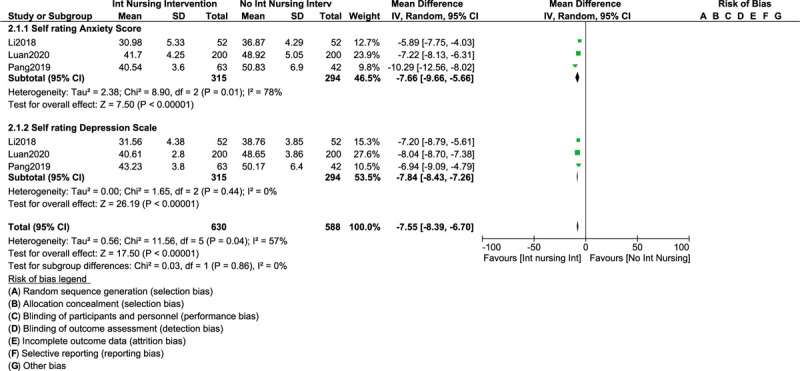
Improvement in self-rating anxiety and depression scales following intense nursing care in patients with liver cancers.

This analysis also showed that SAS and SDS were significantly in favor of the participants with intense nursing care with OR: −7.66, 95% CI: [(−9.66) – (−5.66)]; *P* = .00001 and OR: −7.87, 95% CI: [(−8.43) – (−7.26)]; *P* = .00001 respectively as shown in Figure 3.

**Figure 4:**
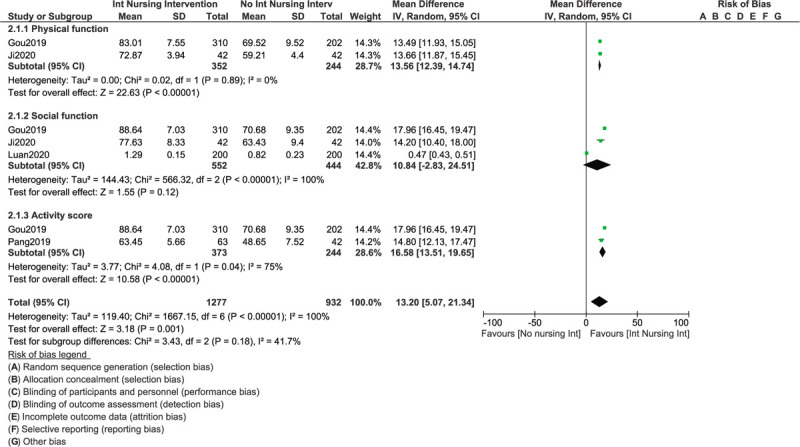
Improvement in other outcomes following intense nursing care in patients with liver cancers.

In addition, physical function (OR: 13.56, 95% CI: 12.39 – 14.74; *P* = .00001), and total activity score (OR: 16.58, 95% CI: 13.51 – 19.65; *P* = .00001) were also significantly in favor of an intense nursing care as shown in Figure 4. However, even if social function (OR: 10.84, 95% CI: −2.83 – 24.51; *P* = .12) was also in favor of an intense nursing care in these patients with liver cancer, the result did not reach statistical significance.

**Figure 5:**
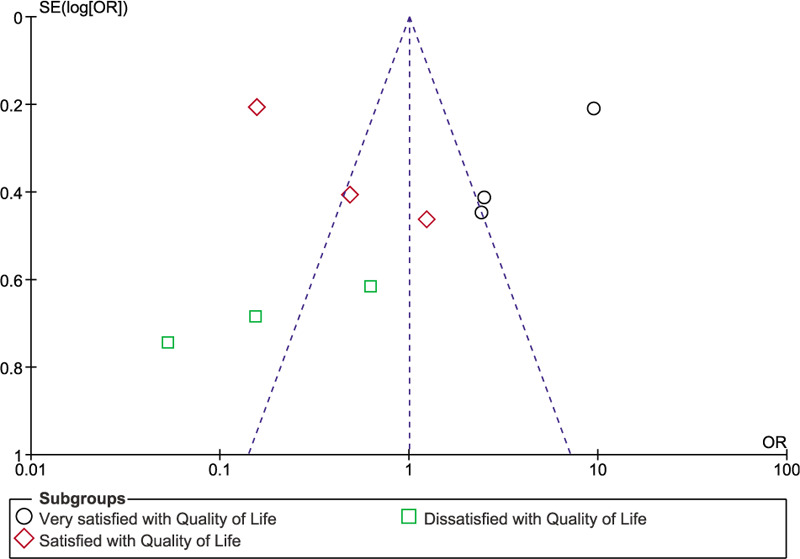
Funnel plot showing publication bias.

Sensitivity analysis showed consistency throughout and publication bias was represented in Figure 5.

## 4 Discussion

Our results showed that intense nursing care in these patients with liver cancer significantly improved quality of life since many participants expressed their significant satisfaction. However, a minority of patients were dissatisfied among those who did not receive intensive nursing care following intervention for liver cancer. Results of this analysis also showed significantly higher rate of anxiety and depression among those patients who did not receive intense nursing care following intervention for liver cancer. Physical function and total activity score was also significantly better in those patients who received intensive nursing care.

A study analyzing the effects of comprehensive nursing care applied in interventional therapy for patients with liver cancer showed that the degree of great satisfaction among the patients was with comprehensive nursing care indicating that the latter was superior compared to the conventional nursing care further supporting the results of this current analysis.^[8]^

In another study^[9]^ based on the efficacy of high quality nursing on alleviating adverse reactions and cancer pain and its effect on quality of life of patients with liver cancer after interventional surgery, the authors demonstrated that this can decrease hospitalization following surgery, improve appetite and sleep quality as well as reduce pain and other post-operative adverse effects. The authors also concluded that the quality of life was significantly improved, further supporting the results of this current analysis.

The potential reasons to support an intensive nursing care could be related to the fact that health education could be enhanced, and a reduction in uncertainty about the disease was assured. An intensive nursing care could also be valuable in terms of helping the patients to look for information and motivations related to the potential cure of their disease.^[16]^ Intensive nursing care also included immediate reporting of symptoms and any abnormal report para and post operatively to the concerned physicians which might lead to a fast response, and a rapid treatment so as to avoid complications, and to improve prognosis among the patients.^[17]^

At last, intensive nursing care is a new nursing model, which might have significant clinical implication in the future among patients with liver cancer requiring surgical intervention.

### 4.1 Limitations

The limitations were: due to the publication of only a few original research articles based on nursing care in patients with liver cancers, our analysis included only a limited number of participants. In addition, due to the involvement of only a few studies, data analysis was carried out on subgroups with the inclusion of only 2 or 3 studies in most of the cases. Another limitation could be the fact that data were retrieved from studies which were mainly observational cohorts, which might have been the cause for this high level of heterogeneity among several subgroups assessing corresponding outcomes. In addition, several relevant outcomes were reported only in 1 study, and were unable to be assessed due to a lack of study data for comparison.

## 5 Conclusions

Our current analysis showed that intense nursing care significantly improved anxiety, depression, and quality of life following interventions in patients with liver cancers. Most of the patients with liver cancers who were assigned to an intense nursing care were very satisfied with their quality of life. However, this hypothesis should further be confirmed in larger nursing related studies based on patients with liver cancers.

## Author contributions

The authors QZ, RW and CL were responsible for the conception and design, acquisition of data, analysis and interpretation of data, drafting the initial manuscript and revising it critically for important intellectual content. QZ and RW are the first co-authors and they wrote this manuscript. All the authors agreed and approved the manuscript as it is.

**Conceptualization:** Qiao Zhang, Rong Wan, Changdan Liu.

**Data curation:** Qiao Zhang, Rong Wan, Changdan Liu.

**Formal analysis:** Qiao Zhang, Rong Wan, Changdan Liu.

**Funding acquisition:** Qiao Zhang, Rong Wan, Changdan Liu.

**Investigation:** Qiao Zhang, Rong Wan, Changdan Liu.

**Methodology:** Qiao Zhang, Rong Wan, Changdan Liu.

**Project administration:** Qiao Zhang, Rong Wan, Changdan Liu.

**Resources:** Qiao Zhang, Rong Wan, Changdan Liu.

**Software:** Qiao Zhang, Rong Wan, Changdan Liu.

**Supervision:** Qiao Zhang, Rong Wan, Changdan Liu.

**Validation:** Qiao Zhang, Rong Wan, Changdan Liu.

**Visualization:** Qiao Zhang, Rong Wan, Changdan Liu.

**Writing – original draft:** Qiao Zhang, Rong Wan.

**Writing – review & editing:** Qiao Zhang, Rong Wan.

## References

[1] Yoshimoto S Loo TM Atarashi K Obesity-induced gut microbial metabolite promotes liver cancer through senescence secretome. Nature 2013;499:97–101. 23803760 10.1038/nature12347

[2] Sha M Seogsong J Xia Q Liver transplantation for cirrhotic patients with small cholangiocarcinoma: what are the optimal criteria? Liver Transpl 2020.

[3] Gentry SE Segev DL Restructuring the Organ Procurement and Transplantation Network contract to achieve policy coherence and infrastructure excellence. Am J Transplant 2019;19:1622–7. 30378753 10.1111/ajt.15161 PMC6494733

[4] Hasselgren K Isaksson B Ardnor B Liver resection is beneficial for patients with colorectal liver metastases and extrahepatic disease. Ann Transl Med 2020;8:109. 32175402 10.21037/atm.2019.12.125 PMC7049033

[5] Rashidian N Willaert W Van Herzeele I FULCRUM Research Group. Key components of a hepatobiliary surgery curriculum for general surgery residents: results of the FULCRUM International Delphi consensus. HPB (Oxford) 2020;Feb 11;S1365-182X(20)30030-7. 10.1016/j.hpb.2020.01.011 32060009

[6] Hunt L Advanced nurse practitioners may have general surgery role. Emerg Nurse 2016;24:8–9. 10.7748/en.24.2.8.s8 27165378

[7] Jennings FL Mitchell M Intensive care nurses’ perceptions of Inter Specialty Trauma Nursing Rounds to improve trauma patient care-A quality improvement project. Intensive Crit Care Nurs 2017;40:35–43. 28259523 10.1016/j.iccn.2017.01.002

[8] Gou Y Yi J Jiang M Analysis on effects of comprehensive nursing care applied in interventional therapy for patients with liver cirrhosis and liver cancer. Iran J Public Health 2019;48:494–500. 31223577 PMC6570817

[9] Jing J Long S Guoyun Z A study on the efficacy of high-quality nursing on alleviating adverse reactions and cancer pain, and its effect on QOL of patients with liver cancer after interventional surgery. Int J Clin Exp Med 2020;13:925–32.

[10] Shengchao Li Ye Zhou Influence of individual nursing care on postoperative early recovery and negative emotions in primary liver cancer patients. Int J Clin Exp Med 2018;11:4702–8.

[11] Luan L Wang L Observation of nursing effect for patients with primary liver cancer before and after transcatheter arterial embolization. Pak J Pharm Sci 2019;32:2455–8. 31894034

[12] Pang L Wang Y Xing Y Application effects of whole course high-quality nursing on patients with liver cancer during radiotherapy. Iran J Public Health 2019;48:1777–85. 31850254 PMC6908919

[13] Higgins JP Altman DG Higgins JP Cochrane Bias Methods Group; Cochrane Statistical Methods Group. The Cochrane Collaboration's tool for assessing risk of bias in randomised trials. BMJ 2011;343:d5928. 22008217 10.1136/bmj.d5928 PMC3196245

[14] Stang A Critical evaluation of the Newcastle-Ottawa scale for the assessment of the quality of nonrandomized studies in meta-analyses. Eur J Epidemiol 2010;25:603–5. 20652370 10.1007/s10654-010-9491-z

[15] Liberati A Altman DG Tetzlaff J The PRISMA statement for reporting systematic reviews and meta-analyses of studies that evaluate healthcare interventions: explanation and elaboration. BMJ 2009;339:b2700. 19622552 10.1136/bmj.b2700 PMC2714672

[16] Kmiecik J Poli A Brons NH Elevated CD3+ and CD8+ tumor-infiltrating immune cells correlate with prolonged survival in glioblastoma patients despite integrated immunosuppressive mechanisms in the tumor microenvironment and at the systemic level. J Neuroimmunol 2013;264:71–83. 24045166 10.1016/j.jneuroim.2013.08.013

[17] Pires NM Dong T Hanke U Hoivik N Integrated optical microfluidic biosensor using a polycarbazole photodetector for point-of-care detection of hormonal compounds. J Biomed Opt 2013;18:097001. 24002194 10.1117/1.JBO.18.9.097001

